# Total Neoadjuvant Therapy with FOLFOX Followed by Short-Course Radiation in Locally Advanced Rectal Cancer—An Alternative Approach Evaluated in a Single-Center Clinical Trial

**DOI:** 10.3390/jcm15093192

**Published:** 2026-04-22

**Authors:** Janice Zhao, Andrea M. Baran, Larissa K. F. Temple, Wenjia Wang, Jason Zittel, Aram F. Hezel, Erika E. Ramsdale, Nabeel Badri, Maria McGreevy, Haoming Qiu, Daniel Mulkerin, Gahyun Gim, Diana Agostini-Vulaj, Christina Cellini, Fergal J. Fleming, Marcus Smith Noel, Richard Francis Dunne

**Affiliations:** 1 Division of Hematology/Oncology, Department of Medicine, Medical College of Wisconsin, Milwaukee, WI 53226, USA; 2 Wilmot Cancer Institute, University of Rochester Medical Center, Rochester, NY 14642, USA; 3 Department of Surgery, University of Rochester Medical Center, Rochester, NY 14642, USA; 4 Division of Hematology, Department of Medicine, University of Florida, Gainesville, FL 32611, USA; 5 Department of Pathology and Laboratory Medicine, University of Rochester Medical Center, Rochester, NY 14642, USA; 6 Ruesch Center for the Cure of Gastrointestinal Cancers, Lombardi Comprehensive Cancer Center, Georgetown University, Washington, DC 20057, USA

**Keywords:** locally advanced rectal cancer, total neoadjuvant therapy, non-operative management, organ preservation, induction chemotherapy, consolidation short-course radiation therapy

## Abstract

**Background/Objectives**: Total neoadjuvant therapy (TnT) has emerged as a treatment option for locally advanced rectal cancer. Few studies have evaluated specifically the use of chemotherapy and short-course radiation therapy (SCRT) in obtaining a complete clinical response (cCR) or near-complete clinical response (nCR) and offering non-operative management (NOM). This phase II study sequences FOLFOX followed by SCRT with the primary aim of evaluating the rate of cCR or nCR. **Methods**: Treatment-naïve adults with non-metastatic clinical T2-3N0 or T1-3 with N1-2a rectal adenocarcinoma deemed candidates for total mesorectal excision (TME) were eligible for this open-label, single-arm clinical trial. This trial evaluated TnT with 5-fluorouracil, leucovorin and oxaliplatin (mFOLFOX6) followed by SCRT. The primary endpoint was the rate of cCR or nCR. Those with cCR or nCR after TnT were offered NOM and close surveillance; all others underwent TME. Secondary endpoints included 1-year disease-free survival (DFS), overall survival (OS), and R0 surgical resection rate. **Results**: Twelve patients of a planned 40 were enrolled with a median follow-up duration of 4.1 years. The study was closed early after results of the OPRA trial suggested a benefit of sequencing radiation prior to chemotherapy when seeking organ preservation. Four of the twelve patients (33%, 95% CI = (9.9%, 65.1%)) achieved cCR or nCR after TnT and underwent NOM; one patient had local regrowth 5.5 months after the completion of TnT and underwent TME. All four were free of disease at time of analysis. The 1-year DFS was 100%. The median OS was not reached. All surgical resections were R0 with no local recurrence after TME. **Conclusions**: This paper suggests that TnT with FOLFOX followed by SCRT is a safe and effective approach for treating locally advanced rectal cancer. This approach can be considered in select patients. The 33% of patients offered NOM is lower than the published 74% in OPRA, however, suggesting that chemotherapy followed by SCRT may not be the most optimal approach if organ preservation is the primary treatment aim.

## 1. Introduction

Colorectal cancer is the third most common cancer in the United States, and the incidence is rising in adults younger than 50 years of age [1,2,3,4,5]. Approximately 47,000 cases of rectal cancer will be diagnosed in the U.S. in 2025 [1].

Over the past decade, we have seen a significant shift in how locally advanced rectal cancers are treated with the advent of total neoadjuvant therapy (TnT) [6,7,8,9,10,11,12,13]. Additionally, there has been an increase in the utilization of short-course radiation therapy (SCRT), especially at the height of the COVID pandemic to reduce patient healthcare visits and exposures [14,15,16,17,18]. The RAPIDO trial evaluated TnT utilizing neoadjuvant SCRT followed by systemic chemotherapy (capecitabine and oxaliplatin [CAPOX] or FOLFOX [5-fluorouracil, leucovorin and oxaliplatin]) and surgical resection compared to long-course chemoradiation therapy (LCRT) followed by surgery and adjuvant chemotherapy [11]. RAPIDO demonstrated a reduction in disease-related treatment failure and distant metastases with TnT [11,19]. Implementing TnT also results in a greater likelihood of complete clinical response and the potential to avoid the toxicities of surgery with non-operative management [20]. Non-operative management allows for the opportunity for organ preservation without compromising oncologic outcomes [12,21].

Optimizing chemotherapeutic and radiation regimens and their sequencing is essential to achieve the best clinical outcomes for patients with locally advanced rectal cancer. We conducted a single institution clinical trial evaluating the rate of complete or near-complete response to TnT, using induction FOLFOX followed by consolidative SCRT. Patients demonstrating a complete clinical response (cCR) or near-complete clinical response (nCR) to treatment were considered for non-operative management.

## 2. Materials and Methods

### 2.1. Study Design and Participants

This study was an open-label, single arm, phase 2 trial conducted at the Wilmot Cancer Institute, University of Rochester Medical Center (URMC) in Rochester, New York. Patients were enrolled in the study from 7 May 2019 to 22 September 2021. The trial protocol was approved by the institutional review board at the University of Rochester. The study was registered with ClinicalTrials.gov (NCT03781323). All study participants provided written informed consent.

Eligible participants were aged 18 years of age and older with newly diagnosed, untreated, histologically confirmed adenocarcinoma of the rectum clinically staged as T2N0, T3N0, or T1-3 with N1-2a and deemed candidates for total mesorectal excision (TME). Patients were also required to meet the following baseline laboratory parameters: absolute neutrophil count (ANC) of 1500/mm^3^ or greater, platelet count of 100,000/mm^3^ or greater, hemoglobin of 8.0 g/dL or greater, total bilirubin within 1.5 times the upper limit of normal (ULN), total creatinine within 1.5 times ULN, and aspartate transaminase (AST) and alanine transaminase (ALT) within 2.5 times ULN. Comorbidities were permitted.

Exclusion criteria included recurrent or refractory adenocarcinoma, evidence of metastatic disease, low rectal tumors likely to require abdominoperineal resection, prior pelvic radiation, and patients with the history of an arterial thrombotic event within the past six months. Full inclusion and exclusion criteria are available in Appendix A.

### 2.2. Procedures and Pathologic Analysis

All patients underwent pre-treatment evaluation which included physical exam, laboratory testing, digital rectal exam (DRE), proctoscopy/sigmoidoscopy, imaging with CT chest, abdomen and pelvis as well as MRI pelvis, and pathologic assessment of the tumor. As per National Comprehensive Cancer Network (NCCN) guidelines, an initial PET scan was not used for staging.

Patients received an initial six cycles of chemotherapy with mFOLFOX6, which consisted of oxaliplatin 85 mg/m^2^ infusion over 2 h followed by bolus 5-fluorouracil (5-FU) 400 mg/m^2^ with leucovorin 400 mg/m^2^ followed by a 46 h infusion of 5-FU at 2400 mg/m^2^ given every 14 days. After six cycles of chemotherapy, patients underwent an interval assessment which included DRE, endoscopy, CT chest, abdomen, and pelvis, and MRI pelvis. Patients with at least 20% response, as assessed by the investigator based on imaging and endoscopy, received an additional four to six cycles of FOLFOX. Patients with less than 20% response were removed from protocol treatment and proceeded with treatment at the discretion of the treating physician. These patients were followed for outcomes and quality of life. Following the completion of chemotherapy, patients underwent a restaging assessment with repeat imaging, DRE, and endoscopic evaluation.

Laboratory, medication, and event monitoring were performed prior to each cycle of chemotherapy. Adverse events related to therapy were assessed and graded by the investigators as per the Common Terminology Criteria for Adverse Events (CTCAE) version 5.0 [22]. Dose reductions were recommended in the event of grade 2 or greater toxicity.

Following chemotherapy, patients proceeded with SCRT. The timing of initiation of radiation was at the discretion of the treating provider. SCRT consisted of 20 Gy in five fractions delivered over five consecutive weekdays to lymph node regions including the mesorectal, internal iliac, and pre-sacral regions with a simultaneous boost of 25 Gy in five fractions to the grossly involved rectal tumor plus adjacent mesorectum and grossly involved adenopathy.

An evaluation for cCR and nCR was made within 6–12 weeks after the completion of radiation. This included imaging with CT chest, abdomen, and pelvis and MRI pelvis as well as DRE and endoscopy. The criteria for eligibility for non-operative management included a demonstrated cCR or nCR. The complete clinical response was defined as a normal DRE and endoscopic evaluation with normal appearing mucosa or scar without nodularity or ulcerations. Near-complete response was defined as DRE with smooth or minor mucosal abnormalities and endoscopic exam with superficial ulceration or erythematous scar. Those with an incomplete response were recommended to proceed with operative management.

Non-operative management surveillance included a history and physical, laboratory monitoring, endoscopic evaluation, and DRE at 3–6 months, 9–12 months, 15–18 months, 21–24 months, 30 months, 36 months, 42 months, 48 months, 54 months, and 60 months following the completion of TnT. CT chest, abdomen, and pelvis as well as MRI pelvis were obtained at 3–6 months, 9–12 months, 21–24 months, 36 months, 48 months, and 60 months.

Pathologic analysis of the surgical specimens when applicable was performed. The treatment effect was assessed utilizing the modified Ryan Scheme for tumor regression score as modified by the College of American Pathologists. According to this scoring system, a score of 0 = complete response (no viable cancer cells), a score of 1 = near-complete response (single cells/rare small groups of cancer cells), a score of 2 = partial response (residual cancer with evident tumor regression, but more than single cells or rare small groups of cancer cells), and a score of 3 = poor or no response (extensive residual cancer with no evident tumor regression) [23].

For assessment of quality of life, patients completed the European Organization for Research and Treatment of Cancer Quality of Life Questionnaire Core-30 (EORTC QLQ-C30) and EORTC Colorectal Cancer Questionnaire (QLQ-CR29) [24,25]. Questionnaires were completed at baseline, interval assessment (after 6 cycles of chemotherapy), restaging assessment (after completion of chemotherapy), and post-radiation assessment. Questionnaires were also completed during the follow-up period after treatment every 6 months during the first two years of follow-up and then at 30 months, 42 months, 48 months, and 60 months. Full quality of life data will be published separately.

### 2.3. Outcomes

The primary endpoint was rate of cCR or nCR. Secondary endpoints included 1-year disease-free survival (DFS), overall survival (OS), R0 surgical resection rate, quality of life, and evaluation of safety and toxicity. DFS was defined as time from treatment initiation to either locoregional failure, metastatic disease, or death. Patients alive and disease free at the end of follow-up were censored at the date of last disease evaluation. OS was defined as the time from treatment initiation to death from any cause with subjects alive at the end of follow-up being censored at date of last contact. As per the OPRA trial, locoregional failure was defined as an unresectable primary tumor after TnT, an R2 resection, or local recurrence after an R0 or R1 resection [12]. Local tumor regrowth in the rectum or regional lymph nodes that occurred in a patient undergoing non-operative management was only considered to be locoregional failure if there was an R2 resection after regrowth. Quality of life was measured using EORTC QLQ-30 and EORTC QLQ-CR 29. Changes in quality of life from baseline were described using means and standard deviations at each time point assessed.

### 2.4. Statistical Analysis

We hypothesized that 30% of patients treated with FOLFOX and SCRT would have a cCR or nCR at time of post-radiation assessment. Given this estimate, we aimed to accrue 40 participants, as a sample size of 40 would produce a one-sided 95% lower-limit exact binomial confidence interval with a width of 13.4% (lower limit of 16.6%). The maximum width of a one-sided 95% lower-limit confidence interval with 40 participants would be 16.1% when the response rate is 50%. We targeted the lower limit of the confidence interval to evaluate whether this treatment regimen demonstrated a sufficiently high estimate of minimal efficacy to warrant further study. Response rates with associated 95% exact binomial confidence intervals are reported.

Survival outcomes are summarized using the Kaplan–Meier method. Adverse events were tabulated by subject and summarized using counts and proportions.

Data from the EORTC QLQ-C30 and QLQ-CR29 were analyzed and scored as per the EORTC scoring guidelines [26]. Missing data were addressed according to the EORTC guidelines. Mean scores for global health status/quality of life and functional scales at baseline (prior to treatment initiation) and at two-year follow up were compared using paired *t*-tests. All analyses were performed using SAS v9.4 (SAS Institute, Inc., Cary, NC, USA).

## 3. Results

### 3.1. Enrollment, Demographics, and Clinical Characteristics

**Figure 1:**
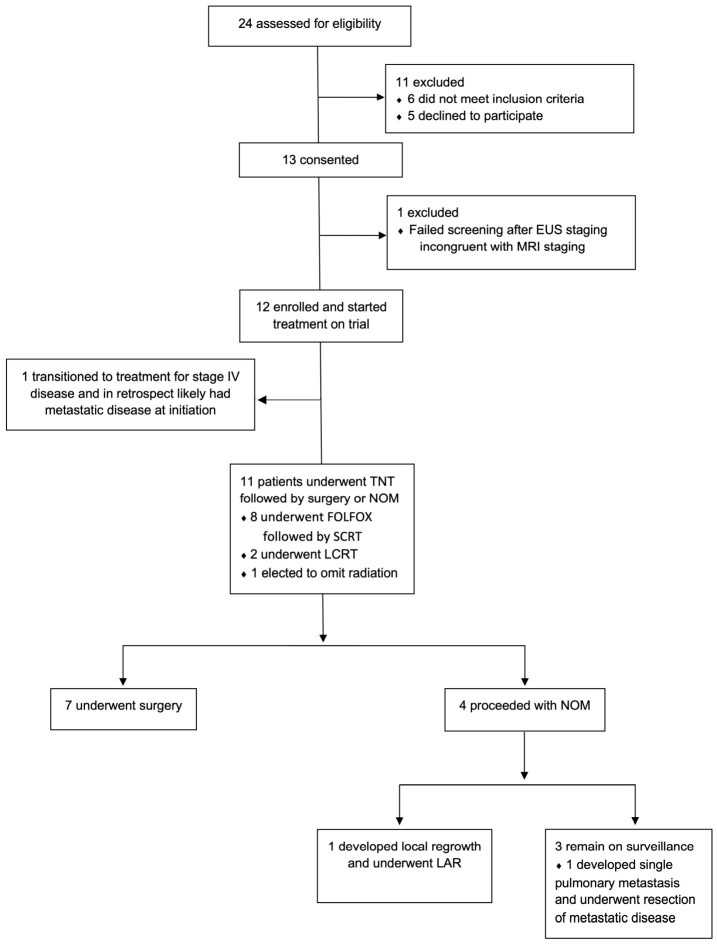
CONSORT diagram. FOLFOX, 5-fluorouracil, leucovorin and oxaliplatin; SCRT, short-course radiation therapy, LCRT, long-course chemoradiation therapy; NOM, non-operative management; LAR, low anterior resection.

Twelve patients ranging in age from 33 to 70 years old were enrolled in the study (Figure 1). The median tumor distance from the anal verge was 10 cm with only one patient having a tumor within 5 cm from the anal verge. Nodal disease was highly prevalent; 75% of patients had at least cN1 disease. Baseline demographics and clinical characteristics are shown in Table 1. Enrollment closed early due to the change in standard of care after the OPRA trial reported higher rates of organ preservation in patients who received TnT with radiation prior to chemotherapy.

### 3.2. Treatment

All twelve patients received chemotherapy with FOLFOX, and eight went on to receive SCRT following chemotherapy (Table 2). One of the eight patients who received SCRT had been removed from the study early due to lack of response to FOLFOX but ultimately elected to receive SCRT due to patient preference and clear circumferential margins.

Four patients did not receive SCRT. One patient with mismatch repair deficient disease elected to omit all radiation and proceeded to surgery after chemotherapy. A second patient in retrospect likely had metastatic disease at the time of treatment initiation. Indeterminate lung nodules at enrollment evolved and were ultimately diagnosed as metastatic disease, and radiation was omitted from the treatment regimen. Two additional patients did not receive SCRT after coming off protocol treatment: one due to lack of response and the other due to disease-related bowel perforation. Both patients received LCRT at the discretion of the treating physician.

All eight patients who received SCRT completed their course of radiation. The time between chemotherapy and radiation for patients who underwent chemotherapy followed by SCRT ranged from five weeks to eleven weeks. The median time to treatment completion, defined as the time from the first dose of chemotherapy to either last radiation treatment if the patient underwent initial non-operative management or surgical date if the patient underwent resection, was 8.2 months (range 6.0–12.2 months). The median time from radiation completion to treatment response evaluation by flexible sigmoidoscopy for those who underwent chemotherapy followed by radiation was 10.4 weeks (range 5.9–14.1 weeks).

### 3.3. Efficacy and Survival Outcomes

At time of data cutoff (5 November 2024), the median duration of follow-up was 4.10 years. The rate of cCr or nCR was 33% (95% CI = 9.9%, 65.1%) with three of the twelve patients achieving a cCR after TnT and one with nCR. The three patients with cCR and one patient with nCR underwent the non-operative management surveillance protocol. The patient with nCR had local regrowth 5.5 months after the completion of SCRT and ultimately proceeded to low anterior resection (LAR). This patient remains disease-free 43 months post-surgery. The other three patients continue on non-operative management surveillance of their rectal primary.

All but one patient who underwent surgical resection of their tumor underwent LAR. The one patient who did not undergo LAR elected to omit radiation treatment and underwent total proctocolectomy given underlying Lynch syndrome. All surgical resections were R0 with no patients experiencing local recurrence after TME at the time of analysis. In an intention-to-treat analysis of all patients in the study who ultimately underwent tumor resection, seven of the tumors were found to have a Ryan score of 3, indicating no evident tumor regression.

**Figure 2:**
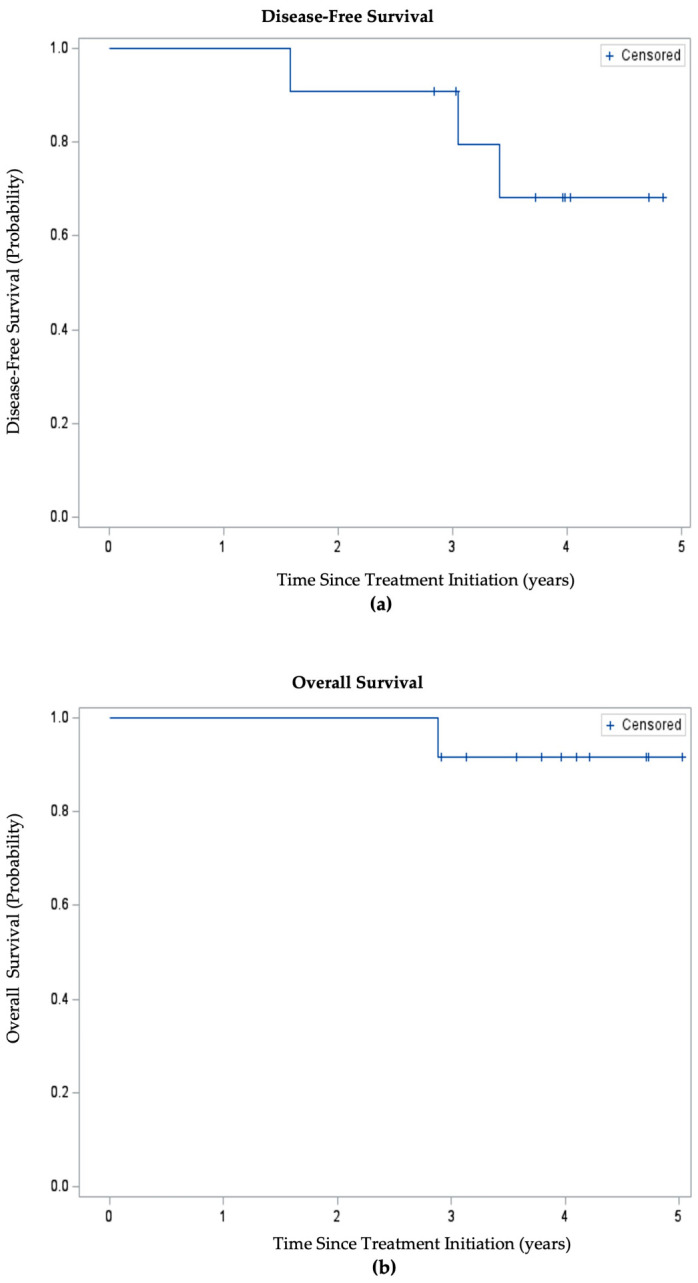
Kaplan–Meier estimates of (**a**) disease-free survival; (**b**) overall survival.

At the time of analysis, the 1-year DFS was 100% and the 3-year DFS was 90.90% (10/11 patients). The patient who in retrospect likely had metastatic disease at the time of treatment initiation was excluded from DFS analysis. Median disease-free survival had not been reached at time of data analysis (Figure 2). At the time of data analysis, 91.67% (11/12) of patients enrolled were alive, and the median OS had not been reached. The one patient who had died by time of analysis was the patient who likely had metastatic disease at the time of treatment initiation. Three additional patients developed metastatic disease during the study. Two of these patients had undergone TME, and one was on the non-operative management surveillance protocol. Two patients, one treated with TME and one managed non-operatively, developed an isolated lung metastasis and subsequently underwent resection of their oligometastatic disease. Both remain without evidence of disease since. The third patient developed unresectable metastatic disease.

### 3.4. Safety

The most common adverse events that occurred during TnT and deemed to be at least possibly related to treatment were fatigue, constipation, and nausea, each affecting 75% of patients (Table 3). The rate of grade 1–2 neuropathy was 66.67%. There was no grade 3 or higher neuropathy. Five grade 3 or higher adverse events occurred. This included two patients with neutropenia (one grade 3 and one grade 4), one patient with colitis (grade 3), and one patient who had sepsis (grade 4) and an acute kidney injury (grade 4).

### 3.5. Quality of Life

**Figure 3:**
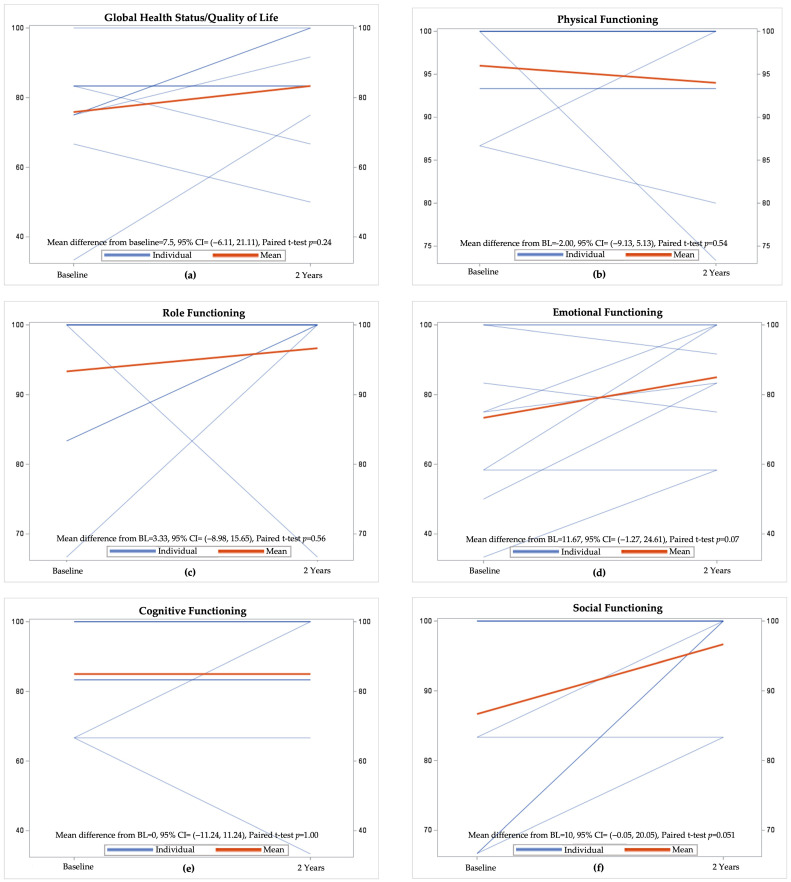
Comparison of baseline and two-year follow-up EORTC QLQ-C30 global health status/quality of life and functional domain scores: (**a**) global health status/quality of life; (**b**) physical functioning; (**c**) role functioning; (**d**) emotional functioning; (**e**) cognitive functioning; (**f**) social functioning.

Ten patients completed EORTC QLQ-C30 prior to treatment initiation (baseline) and at two-year follow-up. When comparing the changes in the global health status/quality of life, physical functioning, role functioning, emotional functioning, cognitive functioning, and social functioning of patients between baseline and two-year follow-up, there was insufficient evidence of a difference in any quality of life measure (Figure 3). Mean scores for global health status and the functional domains remained stable or showed slight improvement from baseline to two-year follow up, except for physical functioning, which showed a numerical decline, though this was not statistically significant (*p* = 0.54). Emotional functioning (*p* = 0.07) and social functioning (*p* = 0.051) demonstrated trends toward improvement.

## 4. Discussion

To our knowledge, this is the first prospective study to report the rate of cCR or nCR and non-operative management with the induction of FOLFOX followed by consolidative SCRT as TnT. The rates of DFS and OS suggest that TnT with FOLFOX followed by SCRT is an effective approach in treating locally advanced rectal cancer. In total, four of twelve patients (33%) experienced a cCR or nCR. Local control was excellent, with no local recurrences observed following TME, including when TME was performed after local tumor regrowth during non-operative management. Additionally, 25% of the patients have sustained rectal organ preservation at the time of data analysis. Of the patients enrolled in the study, 83.33% (10/12) of patients are currently disease-free with a median follow-up of over four years.

Very few studies have reported on the use of SCRT in the treatment of locally advanced rectal cancer particularly with an evaluation for non-operative management. One single-center observational study compared organ preservation with LCRT versus SCRT in patients receiving TnT [27]. In this study, the majority of patients received induction chemotherapy with FOLFOX or CAPOX, although some patients received induction radiation. There were similar rates of eligibility for non-operative management after TnT across radiation modalities with 44.5% in the LCRT cohort and 43.4% in the SCRT cohort eligible for non-operative management. Additionally, rates of distant recurrence, DFS, and OS were comparative among patients on non-operative management. However, patients who had received LCRT prior to non-operative management had higher 2-year organ preservation rates compared to those treated with SCRT (89% vs. 70% respectively, *p* = 0.005). The study also observed higher rates of organ preservation among those who received induction radiation compared to induction chemotherapy in both the LCRT and SCRT TnT cohorts [27]. The results from this observational study as well as the published OPRA trial rate of 74% of patients offered non-operative management (higher than the 33% of patients in our study) suggest that LCRT may be associated with greater rates of organ preservation [12,27,28].

While the use of LCRT may result in higher rates of organ preservation, the data from our study show that cCR and organ preservation can be achieved with a TnT approach of FOLFOX followed by SCRT. Thus, this approach may be an appealing treatment option for select patients, such as those who may face challenges with the time commitments of LCRT. Furthermore, this TnT regimen can be considered in those who may benefit from receiving chemotherapy as opposed to radiation first, such as in certain patients for whom there is concern about bulky tumors or the potential for nodal or metastatic disease.

Research investigating the most optimal TnT regimen for achieving organ preservation is ongoing. The Janus Rectal Cancer Trial is evaluating chemotherapy intensification with FOLFIRINOX (5-fluorouracil, leucovorin, irinotecan, and oxaliplatin) versus FOLFOX or CAPOX after LCRT in TnT for the treatment of locally advanced rectal cancer [29]. Meanwhile, the ACO/ARO/AIO-18.1 study is comparing induction SCRT versus LCRT in TnT with organ preservation being the primary endpoint [30]. This prospective randomized study aims to provide clearer data to support a preferred radiation regimen more definitively.

There were several limitations to our study. First, it was a non-randomized single institution study with a small sample size of 12 patients. Although we initially intended to enroll 40 patients, enrollment was closed early due to evolving standards of care in regard to TnT sequencing for locally advanced rectal cancer [12]. Additionally, due to the use of SCRT in this paper, the decision was made to exclude patients with low rectal tumors and T4 tumors. As previously discussed, the rate of patients offered non-operative management in this paper was lower compared to other studies such as OPRA, which was likely due to differences in treatment sequencing. Institutional differences in determining eligibility for non-operative management based on cCR and nCR may have also played a role.

## 5. Conclusions

This paper suggests that TnT with FOLFOX followed by SCRT is a safe and effective approach for treating locally advanced rectal cancer. This approach can be considered for select patients, and patient priorities should be carefully considered when determining the TnT regimen. However, if organ preservation is a primary treatment goal, our findings suggest induction FOLFOX followed by consolidative SCRT may not be the most optimal approach. Further more definitive studies are ongoing to determine how to best achieve organ preservation.

## Figures and Tables

**Table 1:** Baseline demographics and clinical characteristics.

Median age at enrollment, years (IQR)	54 (41–59)
Number of males	7 (58.3%)
Median baseline CEA, ng/mL (IQR)	1.85 (1.10–10.35)
Median tumor distance from anal verge, cm (IQR)	10 (9–11)
Clinical T stage	*n* = 12
cT2	4 (33.3%)
cT3	8 (66.7%)
Clinical N stage	*n* = 12
cN0	3 (25%)
cN1	3 (25%)
cN2	6 (50%)

**Table 2:** Summary of treatment.

Number of patients who received FOLFOX followed by SCRT	8/12 (66.7%)
Median number of cycles of chemotherapy received	10
Number of patients who received ≥10 cycles of chemotherapy in TnT	9/12 (75%)
Number of patients who underwent SCRT and received complete 25 Gy	8/8 (100%)

**Table 3:** Adverse events of any grade at least possibly related to TnT that occurred in ≥10% of patients.

Adverse Event	Grade 1–2	Grade 3–5
Fatigue	9 (75%)	0 (0%)
Constipation	9 (75%)	0 (0%)
Nausea	9 (75%)	0 (0%)
Neuropathy	8 (66.67%)	0 (0%)
Diarrhea	8 (66.67%)	0 (0%)
Thrombocytopenia	5 (41.67%)	0 (0%)
Dizziness	4 (33.33%)	0 (0%)
Mucositis	4 (33.33%)	0 (0%)
Neutropenia	1 (8.33%)	2 (16.67%)
Vomiting	3 (25%)	0 (0%)
Rash	2 (16.67%)	0 (0%)
Anemia	2 (16.67%)	0 (0%)
Infection	2 (16.67%)	0 (0%)
Dysgeusia	2 (16.67%)	0 (0%)
Thromboembolic event	2 (16.67%)	0 (0%)
Palmar–plantar erythrodysesthesia	2 (16.67%)	0 (0%)
Dry mouth	2 (16.67%)	0 (0%)
Abdominal pain	2 (16.67%)	0 (0%)
Transaminitis	2 (16.67%)	0 (0%)

## Data Availability

The original contributions presented in this study are included in the article. Further inquiries can be directed to the corresponding author.

## Abbreviations

The following abbreviations are used in this manuscript:

5-FU	5-fluorouracil
ALT	Alanine transaminase
ANC	Absolute neutrophile count
AST	Aspartate transaminase
CAPOX	Capecitabine and oxaliplatin
cCR	Complete clinical response
CTCAE	Common Terminology Criteria for Adverse Events
DFS	Disease-free survival
DRE	Digital rectal exam
EORTC QLQ-C30	European Organization for Research and Treatment of Cancer Quality of Life Questionnaire Core-30
FOLFIRINOX	5-fluorouracil, leucovorin, irinotecan, and oxaliplatin
FOLFOX	5-fluorouracil, leucovorin, and oxaliplatin
LAR	Low anterior resection
LCRT	Long-course chemoradiation therapy
nCR	Near-complete clinical response
NOM	Non-operative management
OS	Overall survival
QLQ-CR29	European Organization for Research and Treatment of Cancer Colorectal Cancer Questionnaire
SCRT	Short-course radiation therapy
TME	Total mesorectal excision
ULN	Upper limit of normal
URMC	University of Rochester Medical Center

## Appendix A

Inclusion criteria for the study included the following:

1. Eighteen years of age or older at time of diagnosis;
2. Histologically confirmed adenocarcinoma of the rectum from biopsy;
3. Clinical staging including T2N0, T3N0 tumors, or T1-3 with N1-2a disease;
4. Must be candidates for total mesorectal excision;
5. Treatment naïve for rectal cancer;
6. No history of prior pelvic radiation;
7. No active infections requiring intravenous antibiotics within 14 days of initiating treatment;
8. Meet baseline laboratory parameters: absolute neutrophil count of 1500/mm^3^ or greater, platelet count of 100,000/mm^3^ or greater, hemoglobin of 8.0 g/dL or greater, total bilirubin within 1.5 times the upper limit of normal (ULN), total creatinine total bilirubin within 1.5 times ULN, and aspartate transaminase and alanine transaminase within 2.5 times ULN;
9. Women of childbearing potential, defined as those biologically capable of becoming pregnant, must be negative for pregnancy testing (urine or blood) and agree to use effective contraception starting after negative pregnancy testing and extending to one year after end of treatment;
10. Those who do not read or understand English are eligible and may be consented according to institutional and federal regulations.

Exclusion criteria for the study included the following:

1. Recurrent or refractory rectal adenocarcinoma;
2. T1N0, T4a, T4b, or N2b tumors;
3. Lower rectal cancer requiring abdominoperineal resection;
4. Any evidence of metastatic disease;
5. Primary unresectable rectal cancer. A tumor will be considered unresectable when invading adjacent organs such that an en bloc resection cannot achieve negative margins;
6. Patients unable to undergo MRI imaging;
7. History of any arterial thrombotic event within the past 6 months including angina, myocardial infarction, transient ischemic attack, or cerebral vascular accident;
8. Cardiovascular, hepatic, or renal systemic diseases that would preclude use of chemotherapy per the treating investigator;
9. Peripheral neuropathy > grade 1 by Common Terminology Criteria for Adverse Events;
10. Must not be on any clinical trials involving other experimental therapies before or during study treatment;
11. Women who are currently pregnant or breast-feeding;
12. Patients who are actively trying to father/conceive children;
13. Any other concurrent medical or psychiatric condition deemed inappropriate for entry into the study per the investigator;
14. History of other invasive malignancy within the past three years, except for adequately treated non-melanoma skin cancer, ductal carcinoma in situ of the breast, bladder carcinoma in situ or carcinoma in situ of the cervix.
